# Restrictive Oxygen Use Did Not Improve Outcomes in the UK ROX Study

**DOI:** 10.1111/aas.70098

**Published:** 2025-07-10

**Authors:** Markus B. Skrifvars, Anders Aneman

**Affiliations:** ^1^ Department of Anaesthesiology and Intensive Care Medicine Helsinki University Hospital and University of Helsinki Helsinki Finland; ^2^ Intensive Care Unit Liverpool Hospital, South Western Sydney Local Health District and Southwestern Clinical School, University of New South Wales Sydney Australia

Delivering supplemental oxygen is undoubtedly the most commonly administered therapy in the care of critically ill patients [1] but, similar to many ubiquitous interventions, the underlying clinical evidence for its use is far from robust. Hypoxia, defined as insufficient tissue oxygenation that may impair organ function, is often a consequence of hypoxaemia—low oxygen levels in the blood. Although definitions vary, an arterial oxygen tension (PaO_2_) less than 8 kPa (60 mmHg) or a peripheral oxygen saturation (SpO_2_) below 90% are commonly used thresholds [2], reflecting the start of the steeper portion of the oxyhaemoglobin dissociation curve. In the presence of hypoxaemia, supplemental oxygen is almost universally administered, despite the absence of randomized controlled trials demonstrating its efficacy in this context [1]. Given the well‐known harmful effects of severe acute hypoxaemia, the practice of oxygen supplementation has become routine even in less severe actual or anticipated hypoxaemia.

The optimal target for oxygen delivery, however, remains uncertain. A liberal approach—frequent in intensive care settings for both invasively and non‐invasively ventilated patients—can result in supranormal blood oxygen levels, that is, hyperoxaemia, and potentially tissue hyperoxia. While experimental studies suggest that hyperoxaemia may be harmful [2, 3], conclusive evidence from clinical trials has been lacking.

Several trials in recent years have compared restrictive oxygen strategies, typically targeting SpO_2_ around 90%, with more liberal strategies targeting 94%–97% or usual care [4, 5, 6]. The recently published UK‐ROX study (*UK Intensive Care Unit Randomized Trial Comparing Two Approaches to Oxygen Therapy*) is by far the largest trial to date comparing a restrictive oxygenation target with usual care in invasively ventilated patients [7]. The study was conducted across 97 ICUs in England, Wales, and Northern Ireland, and enrolled 16,500 adult patients following an unplanned ICU admission and requiring mechanical ventilation within a median of 5 h on the ventilator and 1 day in the hospital. This extraordinary achievement was possible by embedding the study procedures in routine clinical care, and the study design should inspire future ICU trials.

In the intervention arm, the SpO_2_ target was 90%, with recommended alarm limits of 88%–92%. The inspired oxygen fraction (FiO_2_) was to be titrated down to the lowest level that maintained this target. In the usual care arm, oxygen therapy followed standard practice, without any upper SpO_2_ alarm limits. Emergency use of FiO_2_ up to 1.0 was permitted in both groups. The primary outcome was 90‐day all‐cause mortality. Secondary outcomes included long‐term mortality, days alive without organ support, and ICU and hospital length of stay. The outcomes were obtained using established registries, further demonstrating the very efficient design of the study.

Ninety‐day mortality was 35.4% (2908/8211) in the conservative oxygen group compared to 34.9% (2858/8183) in the usual care group (adjusted absolute difference 0.7%, 95% CI: –0.7 to 2.0), a difference that was not statistically significant [7]. The point estimate of the difference using conservative oxygen delivery translates into a number needed to harm (NNH) of 143 (95% CI: Number needed to treat of 143 to NNH 50). Although the study did not include a Bayesian analysis, applying a widely non‐informative prior of no difference based on previous studies [4, 5, 6] to the reported odds ratio suggests that any mortality reduction from targeting SpO_2_ of 88%–92% is unlikely, with a posterior probability of 27% [8]. None of the secondary outcomes were significantly different. Subgroup analyses (including patients with hypoxic‐ischaemic encephalopathy, COVID‐19, sepsis, acute brain injury, and those without these conditions and of different ethnicity) did not reveal any treatment effect heterogeneity, to some extent divergent from previous reports [9, 10, 11]. However, given the small sample sizes in many subgroups, these results are exploratory. A prespecified machine learning technique to assess heterogeneity of treatment effect considering more detailed patient characteristics is underway and will be reported separately. This analytical approach can provide a granular insight into who might benefit—or be harmed—by restrictive oxygen targets using patient‐level data [12].

The obvious strength of the UK‐ROX study is its sample size. It was powered to detect a 2.5% change in outcome with the restrictive oxygen target. This appears more realistic than the effect sizes of previous trials. The large number of patients was conducive to achieving a statistical separation of oxygen exposure, while establishing treatment separation can be challenging in ICU trials [13, 14]. Several limitations of the UK‐ROX trial merit discussion. Of the screened population, more than half were not randomized, despite meeting inclusion criteria and lacking exclusion criteria. In 35% (6589 patients), the exclusion was based on a “clinical decision” with limited further detail provided. It remains unclear how the inclusion of these patients might have influenced the results. Another potential limitation is the relatively modest separation between treatment groups with a debatable pathophysiological importance. The average difference of median FiO_2_ over time was just 4%. The average differences in median SpO_2_ and PaO_2_ were around 2% and 1.1 kPa (8 mmHg), respectively. Although statistically significant, the magnitude of these differences was small. Possible reasons include favoring lower oxygen targets in the usual care group. On the other hand, some patients in the restrictive group received higher FiO_2_ than intended. These findings likely reflect the practical realities of bedside oxygen titration and clinicians' instinctive responses to presumed patient needs, which are impossible to control for in a pragmatic clinical trial.

How should these findings be interpreted more broadly? While the lack of statistical significance might be interpreted as equivalence, the point estimate suggests that restrictive oxygen therapy may even be harmful. Thus, any motivation for its use may be in the context of financial or resource restraints to reduce the need for oxygen supplies. For many clinicians, this rationale will seem insufficient if there is even a modest potential for harm. The UK ROX investigators are planning a health economic evaluation, and that is eagerly awaited.

In terms of clinical practice, the UK‐ROX trial does not support a general recommendation to target an SpO_2_ of 90%–92%. A similar finding was seen in patients with trauma [9]. The results of the ongoing MEGA‐ROX trial are much anticipated [15]. In the meantime, questions remain about how best to tailor oxygen therapy to individual patients. Should we, for instance, account for baseline and pre‐disease oxygenation status more explicitly? Age and chronic lung disease lower the level of normal SpO_2_ values, but in young patients without lung pathology, baseline saturation is likely ≥ 95%. It is unclear why, in the setting of critical illness, we would expect 88%–92% to be universally superior. The exception might be the situation where very high FiO_2_ or potentially harmful mechanical ventilation would be needed to achieve a more normal SpO_2_.

Until then, a conventional oxygen target of 94%–97% appears to be the safer and more appropriate default in the ICU [1]. While the blanket use of 40% FiO_2_ “just in case” is outdated, clinicians should not hesitate to use high FiO_2_ in situations where oxygenation is uncertain or compromised—such as in hemodynamically unstable patients, during airway manipulation or patient transport [16]. We are all too familiar with the devastating consequences of severe hypoxia and the resulting brain injury, which remain irreversible and untreatable.

## Author Contributions

Markus B. Skrifvars wrote the firrst draft of this editorial. The text was revised by Anders Åneman. Both authors approved the final version.

## Conflicts of Interest

The authors declare no conflicts of interest.

## Data Availability

Data sharing not applicable to this article as no datasets were generated or analysed during the current study.
